# Supplementary material for: Adaptive integration of model-based and model-free strategies in human reinforcement learning of reachable space
Source: bioRxiv. 2026 Mar 4:2026.03.02.709046. Preprint. [Version 1] doi: 10.64898/2026.03.02.709046 (PMC13001398; doi:10.64898/2026.03.02.709046)
Supplement: Supplement 1 [file NIHPP2026.03.02.709046v1-supplement-1.pdf]

bioRxiv preprint doi: https://doi.org/10.64898/2026.03.02.709046; this version posted March 4, 2026. The copyright holder for this preprint (which was not certified by peer review) is the author/funder, who has granted bioRxiv a license to display the preprint in perpetuity. It is made available under aCC-BY 4.0 International license.

Supplementary Information

Recoverability tests

We performed parameter recovery for the parameters of the model-based (MB) and model-free (MF) algorithms. Tests were performed separately for the Visual-Haptic and Haptic conditions. For each participant, we ran autonomous simulations with the MB and MF algorithms separately using their best-fit parameters ("true" parameters). Then, we fit the same model (MB or MF) to the simulated data and obtained a set of "recovered" parameters.

Fig. S1a shows the recovered parameters against the true parameters. Each dot represents a single participant. For most model parameters, the recovered parameters were close to the true parameters (the dots are close to the dashed $x = y$ unity line), with high Pearson's r values, showing good recoverability. The λ parameter in the MF algorithm in the Haptic condition, which is the memory decay parameter for eligibility traces, had a relatively low r value. Note that this parameter was close to 1 for all participants, which made it difficult to recover the small between-participant differences. Nevertheless, the absolute value of this parameter was recoverable, i.e., the recovered parameters were also close to 1.

We also performed a model recovery test using the data simulated by the MB and MF algorithms. For each simulated dataset, we performed model comparison between the MB and MF algorithms based on the Bayesian Information Criterion (BIC). For all simulated participant datasets (bars in Fig. S1b), the "true" model that simulated the data was favoured ($|\Delta BIC| > 10$), i.e., correctly recovered.

Finally, we performed an additional model recovery test regarding the hybrid-constant (HC) and hybrid-dynamic (HD) models. This specific test is performed to validate that, apart from differentiating MB behaviour from MF behaviour, whether our modelling approach can further identify the true trend of MF weights across trials at the group level. To this end, we ran autonomous simulations with the HC and HD algorithms separately for each participant using their best-fit parameters. Then, we fit both models (HC or HD) to the simulated data, and compared the models based on the overall BIC (sum of BICs across simulated participant datasets). For both the Visual-Haptic and Haptic conditions, the "true" model that simulated the data was favoured ($|\Delta BIC| > 10$), i.e., the group-level trend of MF weight across trials was qualitatively recovered.

bioRxiv preprint doi: https://doi.org/10.64898/2026.03.02.709046; this version posted March 4, 2026. The copyright holder for this preprint (which was not certified by peer review) is the author/funder, who has granted bioRxiv a license to display the preprint in perpetuity. It is made available under aCC-BY 4.0 International license.

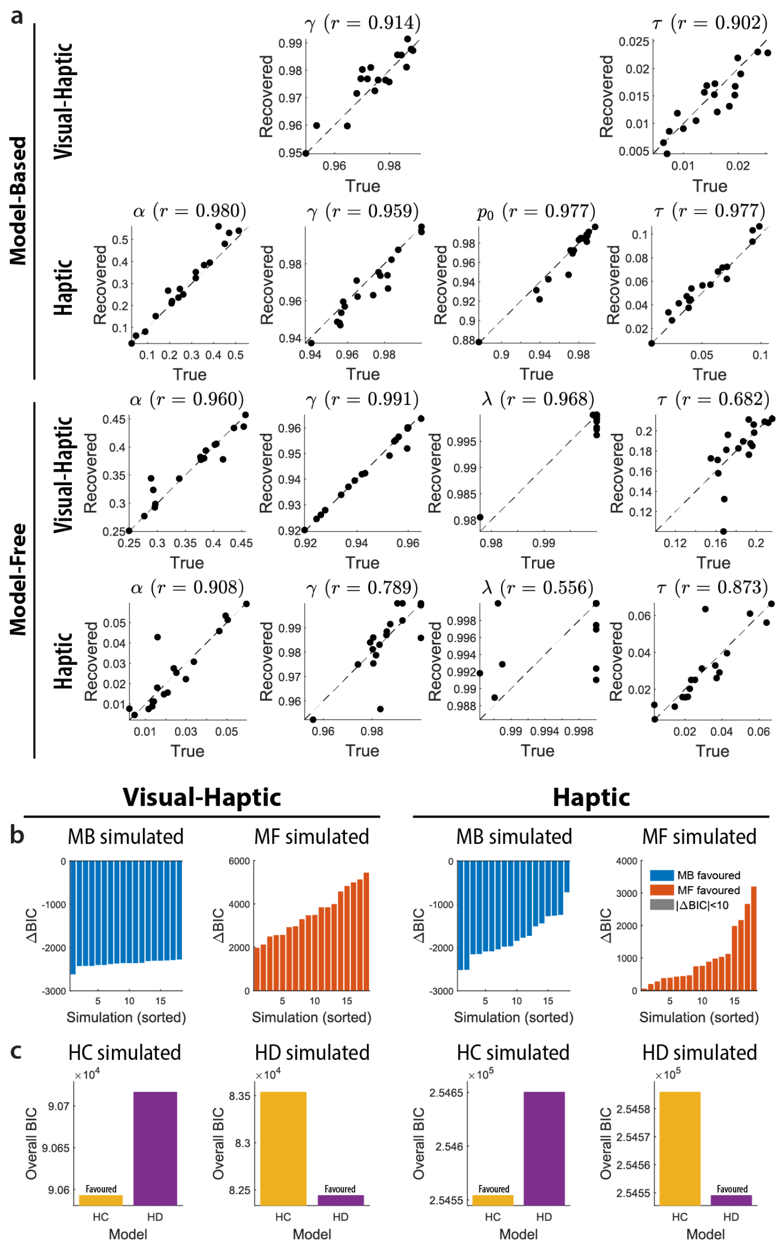

Fig. S1 Model parameters and model identities are reliably recovered from simulated data. a, Parameter recovery for MB and MF algorithms in Visual-Haptic and Haptic conditions. Scatter plots show "recovered" vs. "true" parameters for each participant (dots) relative to the $x = y$ unity line (dashed). b, Model recovery for MB and MF algorithms. Data simulated by each model were fit by both algorithms. The ΔBIC consistently favoured the "true" generating model ($|\Delta BIC| > 10$) across all simulations. c, Group-level recovery for HC and HD models. Overall BIC (summed across participants) favoured the generating model in both experimental conditions. This confirms the modelling approach can correctly identify the trend of MF weights across trials.

bioRxiv preprint doi: https://doi.org/10.64898/2026.03.02.709046; this version posted March 4, 2026. The copyright holder for this preprint (which was not certified by peer review) is the author/funder, who has granted bioRxiv a license to display the preprint in perpetuity. It is made available under aCC-BY 4.0 International license.

Hybrid action strategy divergence analysis

To confirm that the hybrid models meaningfully capture the relative contribution of MB and MF strategies, we compared the policies (probabilities of selecting each available action) generated by the MB and MF components in the hybrid models. The MF weights in the hybrid models are only interpretable when the MB and MF policies are generally distinguishable. Notably, because both components can degrade into a random strategy under specific parametrization (e.g., an extremely high Boltzmann exploration temperature parameter τ), we also compared both components against a random strategy.

We computed the Jensen–Shannon (JS) divergence between MB and MF policies, as well as between each policy and a uniform random policy, at each step. Figure S2 shows the JS divergence between each pair of strategies in each condition as a function of trial number, based on the hybrid-stepwise (HS) model. Results from the other two hybrid models, hybrid-constant (HC) and hybrid-dynamic (HD), are qualitatively similar.

In all three conditions (Visual-Haptic, Haptic, and the navigation task), the MB and MF strategies were consistently different from one another and from a random strategy (JS divergence above zero), for the majority of participants. However, for one participant in the Haptic condition, the MF component converged to a random policy (Fig. S2f). This specific participant exhibited the lowest overall MF weight in the HS model and the second-highest Bayesian evidence favouring an MB strategy over an MF strategy (based on ΔBIC between single MB and MF algorithms) in that condition. These metrics indicate that the hybrid model successfully identified the absence of MF behaviour in this participant; by effectively suppressing the MF weight, the model rendered the component's internal parameters irrelevant to the likelihood function, leading to its "collapse" into a random strategy.

In sum, these results validate the meaningful interpretation of the MB and MF components and their respective weights within the hybrid modelling framework.

bioRxiv preprint doi: https://doi.org/10.64898/2026.03.02.709046; this version posted March 4, 2026. The copyright holder for this preprint (which was not certified by peer review) is the author/funder, who has granted bioRxiv a license to display the preprint in perpetuity. It is made available under aCC-BY 4.0 International license.

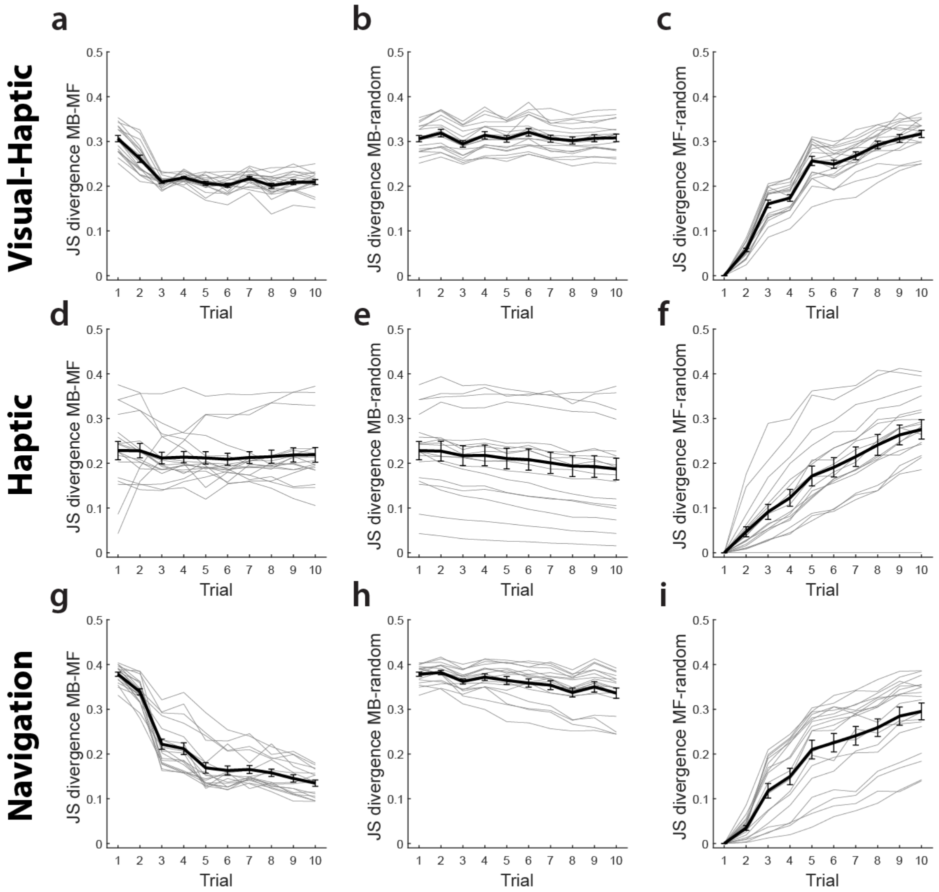

Fig. S2 Model-based and model-free policies are consistently distinguishable within the hybrid framework. a–i, JS divergence across trials for the Visual-Haptic (a–c), Haptic (d–f) conditions in the reachable space task, and the navigation task (g–i). Comparisons include MB vs. MF policies (a, d, g), MB vs. random policies (b, e, h), and MF vs. random policies (c, f, i). Thick black lines represent the group mean, while thin grey lines represent individual participants. Error bars show SEM.

Performance-optimized model-based and model-free algorithms

In Fig. 3m-p, we plotted performance of the MB and MF algorithms simulated using parameters fitted to human participants' data. To evaluate the general performance of the two different algorithms in this maze task, without the constraint that they must produce human-like behaviour, we optimized each algorithm by searching for a set of parameters that minimized the average path length in the simulations. This analysis is based on the Haptic condition, where the maze layout is invisible.

Figure S3 shows the simulation results for the performance-optimized algorithms, together with human performance. The performance-optimized MB algorithm

bioRxiv preprint doi: https://doi.org/10.64898/2026.03.02.709046; this version posted March 4, 2026. The copyright holder for this preprint (which was not certified by peer review) is the author/funder, who has granted bioRxiv a license to display the preprint in perpetuity. It is made available under aCC-BY 4.0 International license.

achieved a 100% success rate and an average path length close to the shortest path length possible under each maze configuration after approximately 2 trials of learning. This demonstrates that the optimized MB algorithm is highly effective at solving our maze learning task, even exceeding average human performance. In comparison, the performance-optimized MF algorithm improved across trials but still performed worse than MB or humans, highlighting the generally limited learning capacity of the MF algorithm despite parameter tuning.

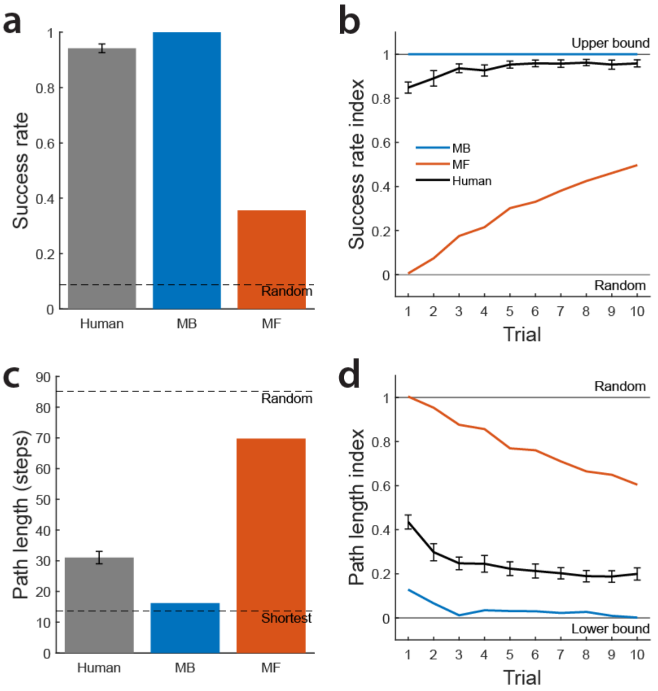

Fig. S3 An optimized model-based algorithm matches or exceeds human performance whereas model-free alone remains limited. a. Overall success rate. b. Success rate index across trials, normalized between random performance (0) and the upper bound (1). c. Average path length in steps. The optimized MB algorithm achieves a path length near the shortest path, while MF remains much higher. d. Path length index across trials. Error bars show SEM across human participants.

Successor representation algorithms

In our 10 × 10 grid maze environment, the successor representation (SR) is defined from a policy-dependent one-step transition matrix T_π and the successor matrix M. Importantly, T_π in the SR algorithm is distinct from the transition function T in our MB algorithm. In the MB algorithm, $T(s, a)$ represents the probability, in the participant's belief, of reaching the next state s' (s' is not blocked) after taking action a from state s, reflecting a mental map of the environment. In the SR algorithm, by contrast, $T_\pi(s, s')$ gives the probability of transitioning to state s' in one step from state s under policy π, reflecting the participant's strategy for choosing actions.

bioRxiv preprint doi: https://doi.org/10.64898/2026.03.02.709046; this version posted March 4, 2026. The copyright holder for this preprint (which was not certified by peer review) is the author/funder, who has granted bioRxiv a license to display the preprint in perpetuity. It is made available under aCC-BY 4.0 International license.

The successor matrix M encodes the expected temporally discounted future occupancies under policy π:

$$M = \sum_{t=0}^{\infty} \gamma^t T_\pi^t = (I - \gamma T_\pi)^{-1} \tag{S1}$$

where t is the number of steps into the future, γ is the temporal discount factor, and I is the identity matrix (with ones on the diagonal and zeros elsewhere).

For each new maze, the initial one-step transition matrix $T_{\pi 0}$ is defined as a uniform random walk over the four possible actions (north, east, south, or west), assuming no maze blocks. For states at edges or corners, probabilities are evenly distributed among valid (i.e., within boundary) neighbours. To account for the assumption that one of the 100 states is absorbing—although its location is unknown at initialization—each row of $T_{\pi 0}$ was scaled to sum to 0.99:

$$T_{\pi 0}(s, s') = \begin{cases} \frac{0.99}{k}, & s' \text{ is one of the } k \text{ valid neighbours of } s \\ 0, & \text{otherwise} \end{cases} \tag{S2}$$

where $k \in \{2, 3, 4\}$ is the number of valid neighbours. The initial successor matrix M_0 is then calculated from $T_{\pi 0}$:

$$M_0 = (I - \gamma T_{\pi 0})^{-1} \tag{S3}$$

In the SR-TD algorithm, the successor matrix M is updated at each step using TD learning with eligibility traces. Specifically, the eligibility trace is initialized over all states at the beginning of each trial:

$$e_0(s) = 0 \tag{S4}$$

and updated at each step t:

$$e_t(s) = \gamma \lambda e_{t-1}(s) + \mathbb{I}(s = s_t) \tag{S5}$$

where λ is the memory decay factor and $\mathbb{I}$ is the indicator function, which takes the value of 1 if the condition holds, and 0 otherwise. The successor matrix M is updated as:

$$M(s, s') \leftarrow M(s, s') + \alpha e_t(s) \Big[\mathbb{I}(s' = s_t) + \gamma M(s_{t+1}, s') - M(s_t, s') \Big] \tag{S6}$$

where α is the learning rate.

The SR algorithm separately encodes the target location with a reward function R:

$$R(s) = \begin{cases} 1, & s \text{ is the target state} \\ 0, & \text{otherwise} \end{cases} \tag{S7}$$

bioRxiv preprint doi: https://doi.org/10.64898/2026.03.02.709046; this version posted March 4, 2026. The copyright holder for this preprint (which was not certified by peer review) is the author/funder, who has granted bioRxiv a license to display the preprint in perpetuity. It is made available under aCC-BY 4.0 International license.

Given the successor matrix M and the reward function R, the value function V for each state s is computed as the expected discounted sum of future rewards:

$$V(s) = \sum_{s'} M(s, s')\, R(s') \tag{S8}$$

During action selection, the algorithm makes decisions based on the value function V. The probability of selecting each action option a_i from the current state is:

$$p(a_i) = \frac{\exp(V(s_i)/\tau)}{\sum_{j=1}^{k} \exp(V(s_j)/\tau)} \tag{S9}$$

where s_i is the intended next state by taking action a_i, k is the number of action options, and τ is the temperature parameter for Boltzmann exploration.

In the SR-MB algorithm, for each new maze, the one-step transition matrix T_π is initialized the same way as in the SR-TD algorithm. In contrast, at each step t, the SR-MB algorithm updates T_π based on the participant's action taken, rather than directly updating the successor matrix M:

$$T_\pi(s_t, s') \leftarrow T_\pi(s_t, s') + \alpha\big[\mathbb{I}(s' = s_{t+1}) - T_\pi(s_t, s')\big] \tag{S10}$$

Thus, T_π estimates the participant's action policy by tracking the historical frequencies of chosen next states.

After updating T_π, the successor matrix is recomputed at each step:

$$M = (I - \gamma T_\pi)^{-1} \tag{S11}$$

This enables M to be globally updated from T_π, implementing MB planning rather than incremental TD updates as in SR-TD.

After computing the successor matrix M, the SR-MB algorithm combines it with the reward function R to obtain the value function V and selects actions based on V using the same procedure as in the SR-TD algorithm.

The SR-TD algorithm has 4 free parameters: α, γ, λ, and τ. The SR-MB algorithm has 3 free parameters: α, γ, and τ. We fit the two algorithms separately for each participant using maximum likelihood estimation (MLE).

Both the SR-TD and SR-MB algorithms we implemented are identical in the Visual-Haptic and Haptic conditions. Specifically, these SR algorithms are strictly policy-based, rather than environment-based as in the pure MB algorithm. Therefore, the SR algorithms can learn only from action experience and cannot use the visual information of the maze layout provided in the Visual-Haptic condition, unlike the MB algorithm.

Fig. S4 compares, in the Visual-Haptic and Haptic conditions, the two SR algorithms with the MB and MF algorithms as well as the three hybrid models (HC, HD and HS) based on the Fixed-Target condition.

Fig. S4a and c show the overall BIC (sum of BICs across all participants) for each model. We did not calculate BIC for the HS model due to its non-parametric nature

bioRxiv preprint doi: https://doi.org/10.64898/2026.03.02.709046; this version posted March 4, 2026. The copyright holder for this preprint (which was not certified by peer review) is the author/funder, who has granted bioRxiv a license to display the preprint in perpetuity. It is made available under aCC-BY 4.0 International license.

regarding the MF weights. For both conditions, the SR-TD algorithm has the highest BIC (i.e., worst) among all models, while the SR-MB lies between the MB and MF algorithms. Neither of the SR models is favoured over the two hybrid models, HC and HD. Fig. S4b and d show the likelihood per step as a function of trials for each algorithm (averaged across different mazes and participants). Both SR algorithms have lower likelihood than the three hybrid models across all 10 trials.

Note that the SR algorithms can be viewed as representation-level hybrid models combining features of MB and MF learning. Our results suggest that such representation-level hybrid models are less capable than our mixture-of-expert hybrid models in terms of explaining participants' behaviour in our tasks.

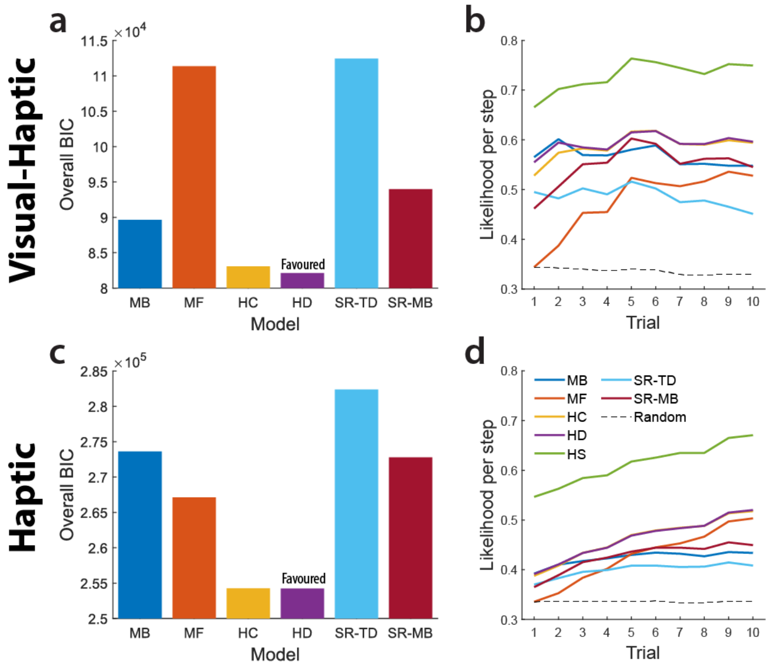

Fig. S4 Successor representation algorithms are outperformed by hybrid models in the reachable space task. a, c, Overall BIC (sum across participants) for the Visual-Haptic and Haptic conditions. The SR-TD algorithm shows the highest (worst) BIC, while the SR-MB model performs between the MB and MF algorithms. Neither SR-based model is favoured over the hybrid-constant (HC) or hybrid-dynamic (HD) models. b, d, Likelihood per step across trials for each algorithm in the Visual-Haptic and Haptic conditions. Both SR models consistently exhibit lower likelihood compared to the three hybrid models (HC, HD, and HS) across all trials.

Potential effect of sex on action strategies

Previous studies have reported sex-related differences in human spatial cognition and navigation strategies. Considering this, we tried to balance the number of female and male participants in our study (Visual-Haptic: 11 females, 7 males; Haptic: 9 females,

bioRxiv preprint doi: https://doi.org/10.64898/2026.03.02.709046; this version posted March 4, 2026. The copyright holder for this preprint (which was not certified by peer review) is the author/funder, who has granted bioRxiv a license to display the preprint in perpetuity. It is made available under aCC-BY 4.0 International license.

9 males). We also performed a two-way ANOVA for unbalanced design to assess the effect of condition (Visual-Haptic vs. Haptic) while accounting for the potential effect of sex on the overall MF weight.

We found a significant main effect of condition ($F(1, 32) = 15.39$, $p = 10^{-4}$), demonstrating that the overall MF weight is significantly higher in the Haptic condition than in the Visual-Haptic condition. Meanwhile, the main effect of sex did not reach the significance level ($F(1, 32) = 3.57$, $p = 0.068$). Moreover, the interaction between condition and sex was non-significant ($F(1, 32) = 2.70$, $p = 0.11$), which statistically confirms that the effect of condition is consistent for both female and male participants. These findings indicate that the observed action strategy difference is a direct result of the condition and is independent of participant sex.

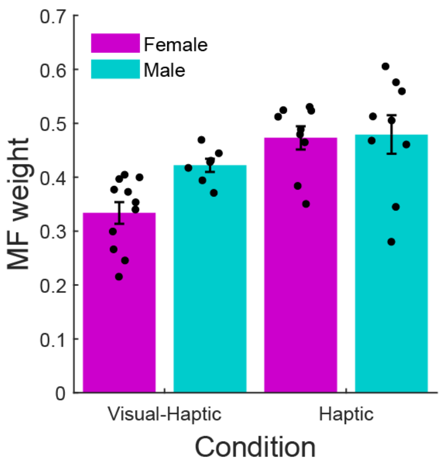

Fig. S5 The condition difference in model-free reliance is independent of participant sex. Bars show overall MF weight, averaged across participants, categorized by sex for the Visual-Haptic and Haptic conditions. Dots represent individual participants, and error bars show *SEM*. While the Haptic condition shows a significantly higher MF weight than the Visual-Haptic condition, the main effect of sex and its interaction with condition were statistically non-significant.
